# Hemorrhagic Stroke Induces a Time-Dependent Upregulation of miR-150-5p and miR-181b-5p in the Bloodstream

**DOI:** 10.3389/fneur.2021.736474

**Published:** 2021-10-27

**Authors:** Pasquale Cepparulo, Ornella Cuomo, Antonio Vinciguerra, Monica Torelli, Lucio Annunziato, Giuseppe Pignataro

**Affiliations:** ^1^Division of Pharmacology, Department of Neuroscience, School of Medicine, University of Naples Federico II, Naples, Italy; ^2^Istituto di Ricovero e Cura a Carattere Scientifico SDN Napoli, Naples, Italy

**Keywords:** microRNA, stroke hemorrhagic, biomarker (BM), rat, blood

## Abstract

To date, the only effective pharmacological treatment for ischemic stroke is limited to the clinical use of recombinant tissue plasminogen activator (rtPA), although endovascular therapy has also emerged as an effective treatment for acute ischemic stroke. Unfortunately, the benefit of this treatment is limited to a 4.5-h time window. Most importantly, the use of rtPA is contraindicated in the case of hemorrhagic stroke. Therefore, the identification of a reliable biomarker to distinguish hemorrhagic from ischemic stroke could provide several advantages, including an earlier diagnosis, a better treatment, and a faster decision on ruling out hemorrhage so that tPA may be administered earlier. microRNAs (miRNAs) are stable non-coding RNAs crucially involved in the downregulation of gene expression *via* mRNA cleavage or translational repression. In the present paper, taking advantage of three preclinical animal models of stroke, we compared the miRNA blood levels of animals subjected to permanent or transient middle cerebral artery occlusion (MCAO) or to collagenase-induced hemorrhagic stroke. Preliminarily, we examined the rat miRNome in the brain tissue of ischemic and sham-operated rats; then, we selected those miRNAs whose expression was significantly modulated after stroke to create a list of miRNAs potentially involved in stroke damage. These selected miRNAs were then evaluated at different time intervals in the blood of rats subjected to permanent or transient focal ischemia or to hemorrhagic stroke. We found that four miRNAs—miR-16-5p, miR-101a-3p, miR-218-5p, and miR-27b-3p—were significantly upregulated in the plasma of rats 3 h after permanent MCAO, whereas four other different miRNAs—miR-150-5p, let-7b-5p, let-7c-5p, and miR-181b-5p—were selectively upregulated by collagenase-induced hemorrhagic stroke. Collectively, our study identified some selective miRNAs expressed in the plasma of hemorrhagic rats and pointed out the importance of a precise time point measurement to render more reliable the use of miRNAs as stroke biomarkers.

## Introduction

Cerebral ischemia results from the interruption of blood flow to a brain region caused by two possible events: a hemorrhagic break or an ischemic occlusion of a cerebral vessel (1, 2). Hemorrhagic stroke accounts for 15% of all strokes, whereas ischemic stroke accounts for 85% of cases. According to the Global Burden of Disease Study, performed from 1990 to 2013, stroke is the second main cause of death (representing 11.8% of all deaths worldwide) and the third leading cause of disability-adjusted life years worldwide (3–6).

To date, the only effective pharmacological treatment for ischemic stroke is limited to the use of recombinant tissue plasminogen activator (rtPA) (7), although endovascular therapy has also emerged as an effective treatment for acute ischemic stroke (8). By contrast, the emergency treatment of hemorrhagic stroke focuses on controlling bleeding and reducing pressure in the brain (1, 2). Unfortunately, the benefit of rtPA treatment for ischemic stroke is time dependent, limited to a 4.5-h therapeutic time window, a largely recognized useful time for penumbra restoring; therefore, the majority of patients cannot benefit from this therapy (9, 10) due to the longer average time needed to carry out an effective diagnosis and therapeutic directioning (11). Most importantly, the use of rt-PA is contraindicated in the case of hemorrhagic stroke, as it would worsen the hemorrhage.

Modern neuroimaging tools, such as computed tomography (CT) or magnetic resonance imaging (MRI), are now used to diagnose a stroke and identify its subtype (12, 13). However, some hospitals do not yet provide MRI and CT service, and many patients cannot benefit from these techniques in the diagnostic process. Moreover, the time required to reach the medical center and to prepare the patients for imaging tests often does not match with the urgency of an early diagnosis for an immediate therapy. In addition, the application of these imaging tools weighs on stroke care costs (14). For all these reasons, the search of biomarkers is critically important to speed up the diagnosis of stroke and selectively distinguish cerebral ischemia from hemorrhagic damage in order to ensure therapeutic interventions in a very short time frame from the onset of the disease.

microRNAs (miRNAs) are evolutionarily conserved non-coding RNAs consisting of 20–22 nucleotides (15), crucially involved in the downregulation of gene expression *via* mRNA cleavage or translational repression (16). Over the last 10 years, the role of miRNAs in stroke has been widely discussed and evaluated, focusing attention on the regulation of stroke risk factors (17) and the mechanisms activated and elicited by the ischemic insult (18).

For the first time, in the last decade, miRNAs have been observed outside the cells, including in various body fluids (19). Several release mechanisms have been hypothesized: one of these consists in microRNA release by cells through microvesicles that originate by outward budding and fission of the plasma membrane (20). Moreover, a specific type of vesicle with a characteristic process of biogenesis, called exosomes, is shown to be enriched of miRNA and involved in the phenomena of cell-to-cell communication (21). Alternative mechanisms of miRNA transport concern the activity of apoptotic bodies, formed during the programmed cell death, and high-density lipoproteins. In this scenario, circulating microRNAs become important mediators of cell communication by altering the gene expression of recipient cells, and the modification of their release in biofluids reflects the expression changes occurring in the origin cells (22).

Whatever the origin of miRNA is, the presence of microRNA in blood and the ability to measure their levels in a non-invasive way have opened new doors in the search for peripheral biomarkers for the diagnosis and prognosis of diseases such as brain ischemia. Indeed the expression levels of miRNAs in blood are reproducible and indicative of several diseases (23). Since the recommended therapeutic window is very limited, the biomarkers for stroke have the potential to expedite diagnosis and the institution of treatment.

In the present comparative report, the plasma microRNA levels were assessed in both rat models of ischemic and hemorrhagic stroke in order to characterize a specific signature of microRNA potentially useful to distinguish among stroke subtypes.

## Methods

### Animals

Male Sprague–Dawley rats (Charles River), weighing 200–250 g, were housed under diurnal lighting conditions (12 h darkness/light) and in a conditioned room (23°C). The experiments were performed according to the international guidelines for animal research and approved by the Animal Care Committee of “Federico II,” University of Naples, Italy. The animals, during any surgical or invasive procedure, were anesthetized using a mixture of oxygen and sevoflurane at 3.5% (Medical Oxygen Concentrator LFY-I-5A), and the rectal temperature was maintained at 37 ± 0.5°C with a heat-controlled mat (Harvard Apparatus). The rats were randomly assigned into experimental and control groups.

Among the 55 animals used for the present study, seven have been excluded from the statistical analysis. In particular, two died during the surgical procedures of hemorrhage induction, and five were excluded for lack of achievement of stroke models.

### Permanent and Transient Focal Ischemia

Stroke was induced by middle cerebral artery occlusion (MCAO) by introducing a suture filament into the internal carotid artery until the middle cerebral artery (24), modified and readapted in our laboratory (25). Briefly, under an operating stereomicroscope (Nikon SMZ800, Nikon Instruments, Florence, Italy), right carotid bifurcation was identified and exposed by using surgical pincers (Dumont #7, FST), and the external carotid artery near the bifurcation was cut and electrocauterized to create a stump on the artery. A silicon-coated nylon filament (Doccol, CA, USA) was inserted through the stump and gently advanced 19 mm into the right internal carotid artery in order to reach the origin of the middle cerebral artery. All animals were sacrificed after 24 h from MCAO. In the model of transient ischemia, the filament was removed after 100 min, and the animals were sacrificed after 3 and 24 h from reperfusion.

### Intracerebral Collagenase-Induced Hemorrhage

Intracerebral hemorrhagic stroke was induced in rats as previously described (26, 27) and adapted in our laboratory. Briefly, using a stereotaxic apparatus, the anesthetized rats were injected with 0.5 U collagenase 2-μl volume dissolved in phosphate-buffered saline (collagenase type VII from Sigma-Aldrich, catalog number C2399) through a Hamilton syringe (26-gauge needle, 10-μl volume) in a burr hole into the right striatum (3 mm lateral to midline, 0.5 mm anterior to bregma, and 5.2 mm below the surface of the skull) over 5 min. The syringe was kept in place for 10 min and removed over 5 min. The animals were sacrificed after 24 h from collagenase injection.

### Monitoring of Blood Gas Concentration and Cerebral Blood Flow

A catheter was inserted into the femoral artery to measure the arterial blood gases before and after ischemia (Rapid lab 860; Chiron Diagnostic, Medfield, MA, USA). Induction of ischemia was confirmed by monitoring the regional cerebral blood flow (CBF) in the area of the right MCA through a disposable microtip fiber optic probe (diameter, 0.5 mm) applied on the right temporo-parietal region of the skull, connected through a Master Probe to a laser Doppler computerized main unit (PF5001; Periflux system, 5000, Perimed AB, Järfälla, Sweden), and analyzed using PSW Perisoft 2.5 (28). CBF monitoring was continued up to 30 min after the end of the surgical procedure once the occurrence of reperfusion was verified.

### microRNA Expression Profiling by Microarray

Brain regions corresponding to the ischemic core and penumbra were dissected from rats subjected to sham surgery and transient middle cerebral artery occlusion (tMCAO). Total RNA from brain tissues was extracted with Trizol, following the instruction of the supplier (TRI Reagent®-Sigma), and RNA quality was assessed using a Thermo Scientific™ NanoDrop™ One Microvolume UV–vis spectrophotometer. The RNA samples were sent to LC Sciences (Houston, Texas, USA), a global biotechnology company which performed a miRNA microarray analysis, including separation, quality control, labeling, hybridization, and scanning. The array contained 810 mature rat miRNA probes for the whole *Rattus norvegicus* miRNome based on a database of published miRNA sequences and annotation (Sanger miRbase Release 21.0). In detail, hybridization was performed on a μParaflo microfluidic chip using a micro-circulation pump (Atactic Technologies, Inc., Houston, TX, USA). After the hybridization, the chips were washed, and then the fluorescence data images were collected using a laser scanner (GenePix 4000B; Molecular Devices, LLC, Sunnyvale, CA, USA) and digitized using Array-Pro image analysis software (Media Cybernetics, Inc., Rockville, MD, USA). The chips were scanned at a pixel size of 10 μM with Cy3 Gain and Cy5 Gain at 460 and 470 nm scanning, respectively. The data were analyzed by first subtracting the background and then normalizing the signals using a locally weighted regression filter.

The datasets presented in this study can be found in the online GEO dataset repositories.

**Figure 1:**
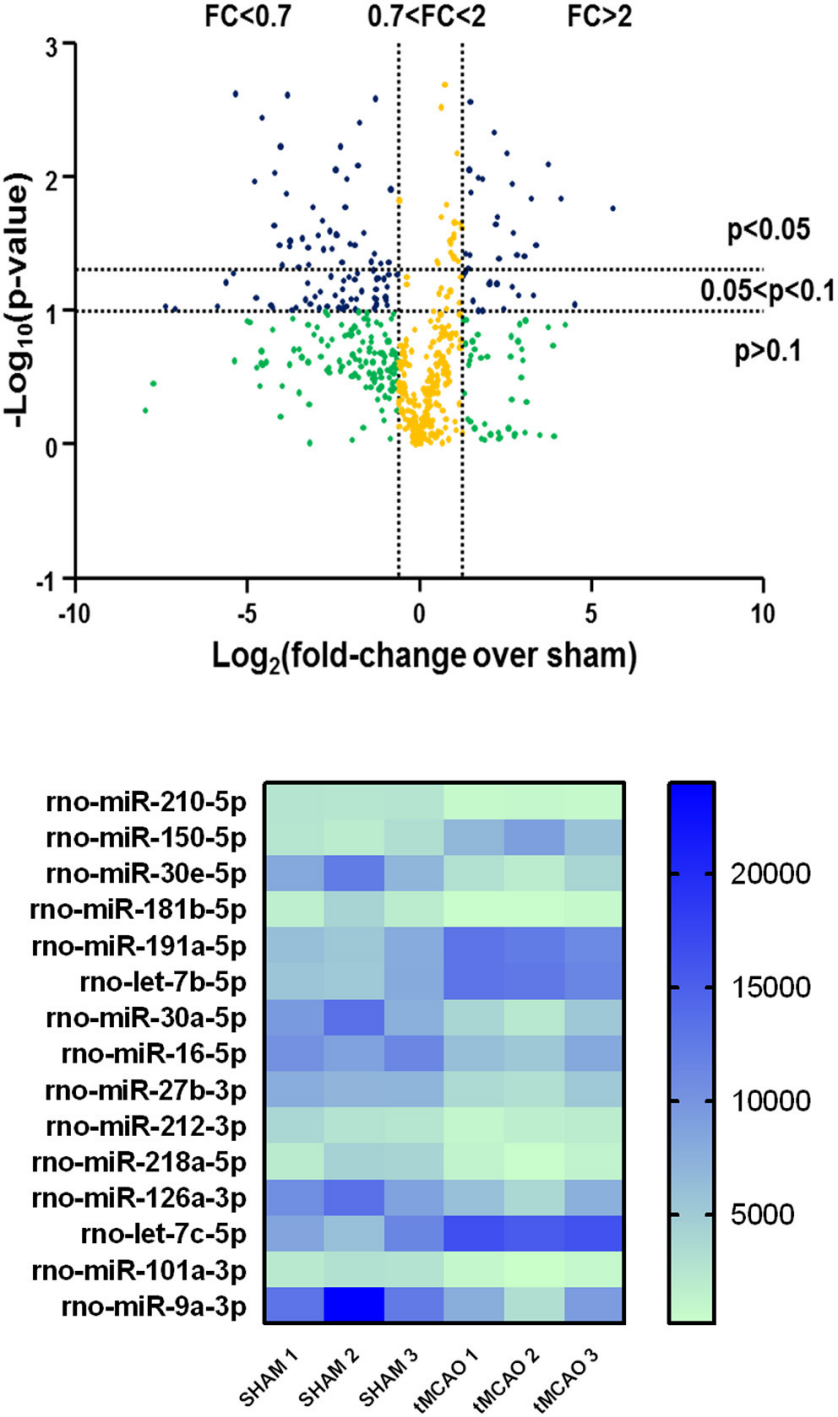
Volcano plot analysis of microRNA expression profiles in the whole ischemic area from sham and transient middle cerebral artery occlusion (tMCAO) animals. Volcano plot analysis in the higher side of the panel shows the miRNA expression changes after tMCAO induction according to a microarray analysis. The y-axis corresponds to the negative log_10_ (*p*-value), and the x-axis displays the log_2_ (fold change) value. The blue dots represent the miRNAs of the tMCAO group with variations of fold change more than 2 or <0.7 compared to the sham group, with a *p* < 0.1. The yellow dots belong to miRNAs whose expression changes were less relevant, with fold change values between 0.7 and 2. The green dots comprise miRNAs with high fold change values but statistically not significant (*p* > 0.1). A heat map of the 15 microRNAs selected from a microarray analysis is shown in the lower side of the panel. The signal intensity values for each significantly expressed miRNA of each sample are reported (*p* < 0.05) in color-coded blocks, according to the colorimetric scale at the right side of the panel.

A significantly different expression pattern between tMCAO and sham-operated groups was obtained by microarray analysis, as shown in the volcano plot graph (Figure 1, at the top). A clustered heat map was then extrapolated, showing a colorful illustration of miRNA expression profiles across three animals for each experimental condition (Figure 1, at the bottom).

### Plasma Sample Collection

Blood samples were withdrawn from the tail vein of anesthetized rats before the ischemia induction and at different time intervals from reperfusion by using a 1-ml syringe with a 23-G needle and collected in BD Vacutainer tubes (K3 EDTA 5.4 mg). To separate the plasma, the blood samples were centrifuged in the same collecting tubes at 1,500 × *g* (2,900 rpm) for 8 min at room temperature in a ALC PK 120 centrifuge. The supernatant plasma was transferred to sterile tube and centrifuged in an Eppendorf centrifuge at 11,000 rpm to purify the sample from any cellular residues. Prior to RNA extraction, the absorbance at 415 nm of a 50-μl aliquot for each sample was measured in a Bio-rad Microplate Reader to evaluate the presence of free hemoglobin because of a previous hemolytic process. The data reported in the literature suggest working on plasma samples with absorbance values below 1.0 OD. This restriction is necessary in order to obtain an evaluation of microRNAs that are present in exosome or free in plasma and released after ischemic lesion, excluding those present in the red and white blood cells and released following hemolysis.

### microRNA Isolation and Assessment From Brain and Plasma Samples

RNA samples were prepared from the ischemic core, corresponding to the striatum, and penumbra region, corresponding to the temporo-parietal brain surrounding cortex. These brain regions were dissected from rats subjected, respectively, to sham surgery and to tMCAO. The sham-operated rats are, in fact, healthy animals. This experimental group has been chosen in order to compare the miRNA expression to that of a control group that underwent similar conditions in terms of anesthesia and rat manipulation. Total RNA from brain tissues was extracted with Trizol (TRI Reagent®-Sigma) following the instruction of the supplier, and RNA quality was assessed using a Thermo Scientific™ NanoDrop™ One Microvolume UV–vis spectrophotometer.

The expression levels of 15 microRNAs selected from the microarray analysis performed on ischemic brain were singularly evaluated by real-time PCR in plasma of rats subjected to permanent or transient focal cerebral ischemia or to intracerebral collagenase-induced hemorrhage (ICH) at different duration times (0.5, 3, and 9 h from ischemic stroke induction).

miRNA isolation from plasma samples was performed with miRNeasy Serum/Plasma Kit (Qiagen) according to the protocol of the manufacturer. For miRNA analysis in the plasma samples, not specific concentration but precise volumes (5 μl) of RNA were retrotranscribed by using High-Capacity cDNA Reverse Transcription Kit (Applied Biosystems) and Taqman probes (Thermo Fisher Scientific), following TaqMan Small RNA Assays Protocol (16°C for 30 min, 42°C for 30 min, and 85°C for 5 min). Quantitative real-time polymerase chain reaction was performed with TaqMan Universal PCR Master Mix II (Applied Biosystems) in a 7500 Fast Real-Time PCR System (AB Applied Biosystems). The cDNA samples were amplified simultaneously in triplicate in one assay run, following the protocol for Taqman assays: 50°C for 2 min, 95°C for 10 min, 40 cycles of amplification of 95°C for 15 s, and 60°C for 1 min. The results were analyzed and exported with 7500 Fast System SDS Software.

The TaqMan probes used are the following: mmu-miR-210^*^ (ID: 462444_mat), hsa-miR-150 (ID: 000473), hsa-miR-30e (ID: 002223), hsa-miR-181b (ID: 001098), hsa-miR-191 (ID: 002299), hsa-let-7b (ID: 002619), hsa-miR-30a-5p (ID: 000417), hsa-miR-16 (ID: 000391), hsa-miR-27b (ID: 000409), mmu-miR-212 (ID: 002551), hsa-miR-218 (ID: 000521), hsa-miR-126 (ID: 002228), hsa-let-7c (ID: 000379), hsa-miR-101 (ID: 002253), hsa-miR-9^*^ (ID: 002231), and miRNA Control Assay U6 snRNA (ID: 001973).

### Information on microRNA-Gene-Disease Ontology Interactions

The extensive file of predicted or verified targets of all aberrantly expressed microRNAs in plasma from rats subjected to brain ischemia of different entity, permanent or transient, or origin, hemorrhagic or occlusive, indicates that a large group of genes may be potentially dysregulated since the prenatal period of life. However, for the present study, the attention has been mainly focused on those genes coding plasma membrane proteins controlling ion influx or efflux, whose regulation has been investigated and acknowledged in stroke mechanisms and in several neuroprotective approaches (Supplementary Table 1). Four different web servers, each one based on a specific algorithm, were used to predict the targets of those microRNAs that have been found to be dysregulated in plasma from rats subjected to brain ischemia of different entity, permanent or transient, or pathophysiology, hemorrhagic or occlusive.

TargetScan is a computational method to predict the targets of conserved vertebrate miRNAs, integrating the model of miRNA–mRNA interaction on the basis of thermodynamics and sequence alignment analysis between miRNA binding sites among different species (29). The latest updated 7.2 version examined the function of non-canonical binding sites identified in recent studies and considers 14 different features of the microRNA, microRNA site, or mRNA to predict those sites within mRNAs that are most effectively targeted by microRNAs (30).

MiRDB is an online database for miRNA target prediction and functional annotations (31). All targets were predicted by a bioinformatics tool, MirTarget, which was developed by analyzing thousands of miRNA-target interactions from high-throughput sequencing experiments (32).

PicTar uses the criteria of co-expression in space and time of miRNAs and their targets through combinations of different microRNAs (33). This algorithm requires that the binding stability of the putative miRNA-target interaction, measured by thermodynamic binding energy, is higher than a specified threshold.

miRmap is an open source software library which employs thermodynamic, evolutionary, probabilistic, or sequence-based features (34), making it currently the most comprehensive miRNA target prediction resource. Only miRNA-mRNA interactions with a miRmap score above 70 were considered for the present study.

### Statistical Analysis

As regard the microarray experiments, Student's *t*-test analysis was conducted for individual comparisons between the two experimental groups. The false discovery rate was *P* < 0.05 and served as the cut-off criteria. The data were log_2_ transformed and median centered by Cluster 3.0 software (Informer Technologies Inc., Los Angeles, CA, USA). Real-time PCR results are expressed as fold change (2^−Δ*ΔCt*^) compared to the control group set to 1, following the instructions provided by the literature (35). Briefly, the difference between the Ct values of the gene of interest and the internal control (ΔCt) is calculated for both control sample and target sample. Then, the difference between the ΔCt of the target sample and the control sample (ΔΔCt) is calculated. The fold change of gene expression of target samples compared to the control sample is calculated as 2^−Δ*ΔCt*^. Values are expressed as means ± standard deviation. Statistical analysis was performed with GraphPad Prism 5.0 (GraphPad Software, Inc., San Diego, CA) using one-way analysis of variance followed by Newman–Keuls *post-hoc* test for groups of more than two. Statistical significance was accepted at 95% confidence level (*p* < 0.05).

## Results

### Circulating miR-16-5p, miR-101a-3p, miR-218a-5p, and miR-27b-3p Are Upregulated in the Plasma of Rats 3 h After MCAO Onset

**Figure 2:**
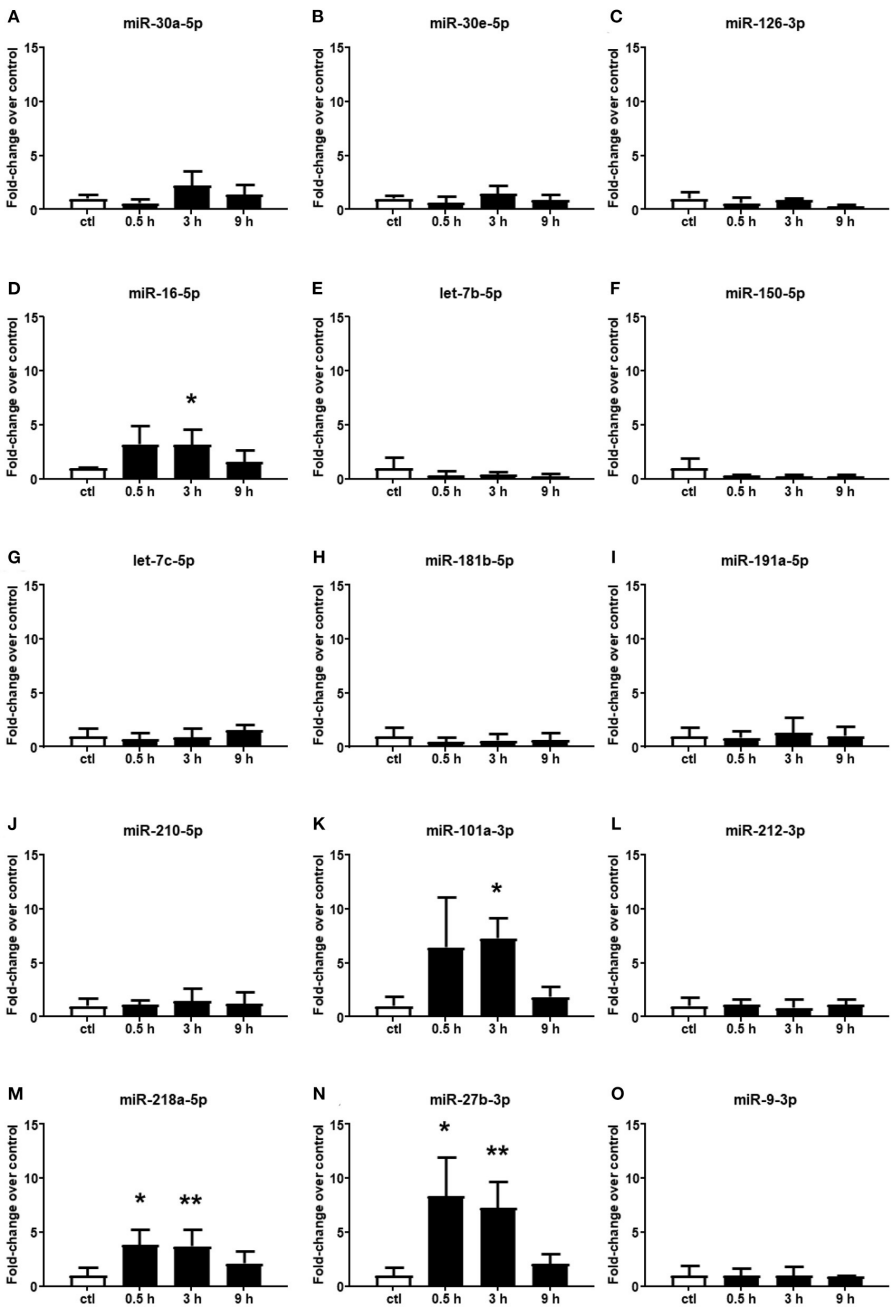
Plasma microRNA expression analysis after permanent middle cerebral artery occlusion by real-time PCR. The microRNA levels analyzed by real-time PCR in plasma samples withdrawn from the tail vein of rats subjected to permanent middle cerebral artery occlusion are expressed as fold change over the control group. **(A–O)** indicate miRNAs evaluated. Each column represents the mean ± SD. The results of the microRNA expression were normalized with respect to U6 snRNA as the internal control. *n* = 3 or 4 per group. *p < 0.05 vs. control group. **p < 0.01 vs. control group.

Among the 15 microRNAs selected from the microarray analysis, 11 miRNAs showed no significant difference in plasma samples at all times assessed after cerebral ischemia compared to the control group (Figures 2A–C,E–J,L,O). By contrast, miR-16-5p, miR-101a-3p, miR-218a-5p, and miR-27b-3p resulted to be upregulated in plasma samples withdrawn up to 3 h of permanent middle cerebral artery occlusion (pMCAO) (Figures 2D,K,M,N). These miRNAs did not display an overexpression after 9 h of permanent ischemia.

### Circulating miR-150-5p, let-7b-5p, let-7c-5p, and miR-181b-5p Are Selective Biomarkers of Hemorrhagic Stroke

**Figure 3:**
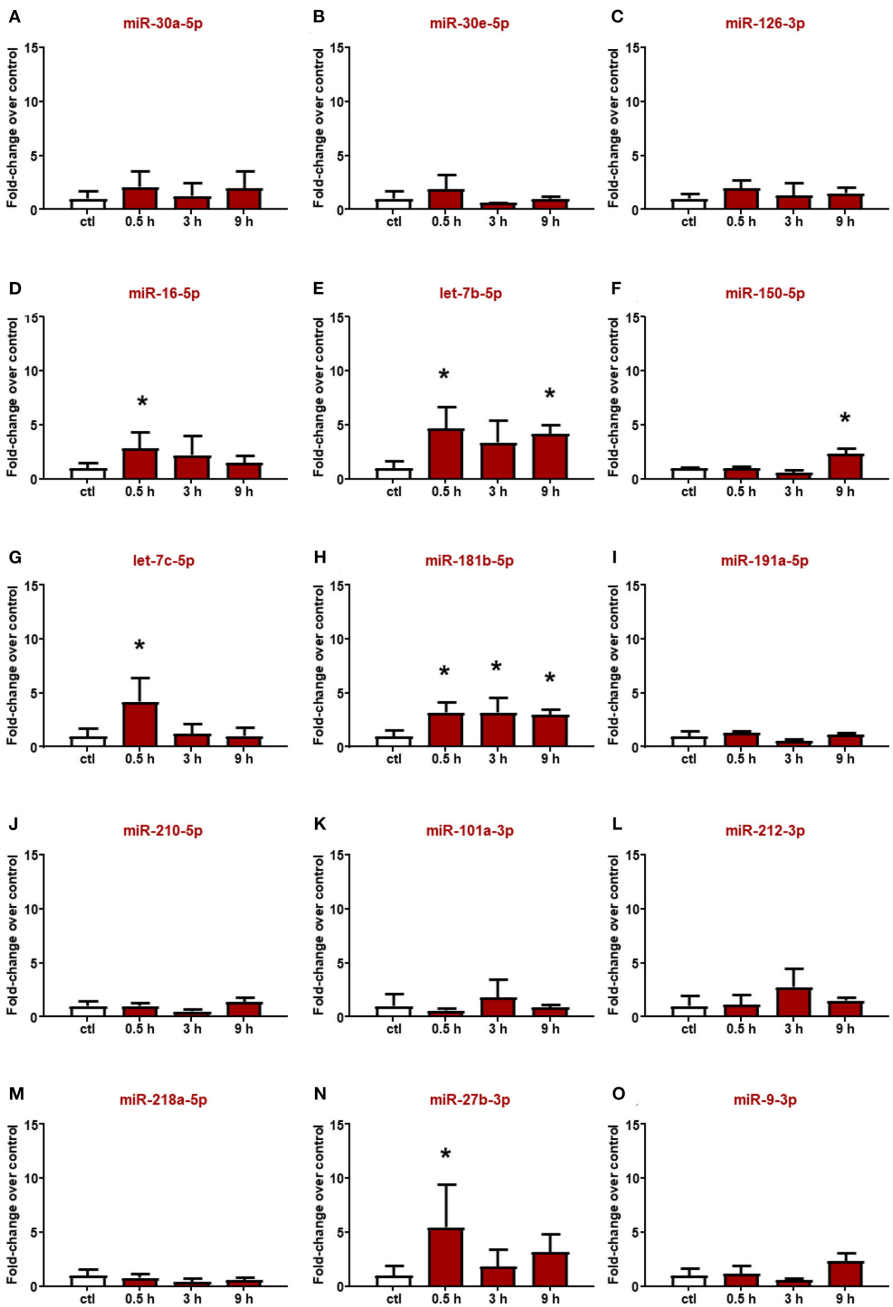
Plasma microRNA expression analysis after intracerebral hemorrhage by real-time PCR. The microRNA levels analyzed by real-time PCR in plasma samples withdrawn from the tail vein of rats subjected to intracerebral hemorrhagic stroke are expressed as fold change over the control group. **(A–O)** indicate miRNAs evaluated. Each column represents the mean ± SD. The results of the microRNA expression were normalized with respect to U6 snRNA as internal control. *n* = 5 or 6 per group. *p < 0.05 vs. control group.

Altered miRNAs in the plasma of rats subjected to ischemic stroke were evaluated also in plasma from ICH rats (Figure 3); among them, miR-16-5p and miR-27b-3p significantly increased 30 min after hemorrhagic stroke, but the levels were already restored at 3 h (Figures 3D,N). Conversely, miR-101a-3p and miR-218a-5p did not show expression changes at any time evaluated (Figures 3K,M). Moreover, some microRNAs whose plasma levels were not modulated by ischemic stroke displayed upregulation after ICH. Indeed let-7c-5p was upregulated only after 30 min from the onset of hemorrhagic stroke (Figure 3G), whereas the let-7b-5p, miR-150-5p, and miR-181b-5p levels significantly increased up to 9 h (Figures 3E,F,H).

### Circulating miR-16-5p and miR-101a-3p Expression in the Plasma of Ischemic Rats Is Affected by Reperfusion

**Figure 4:**
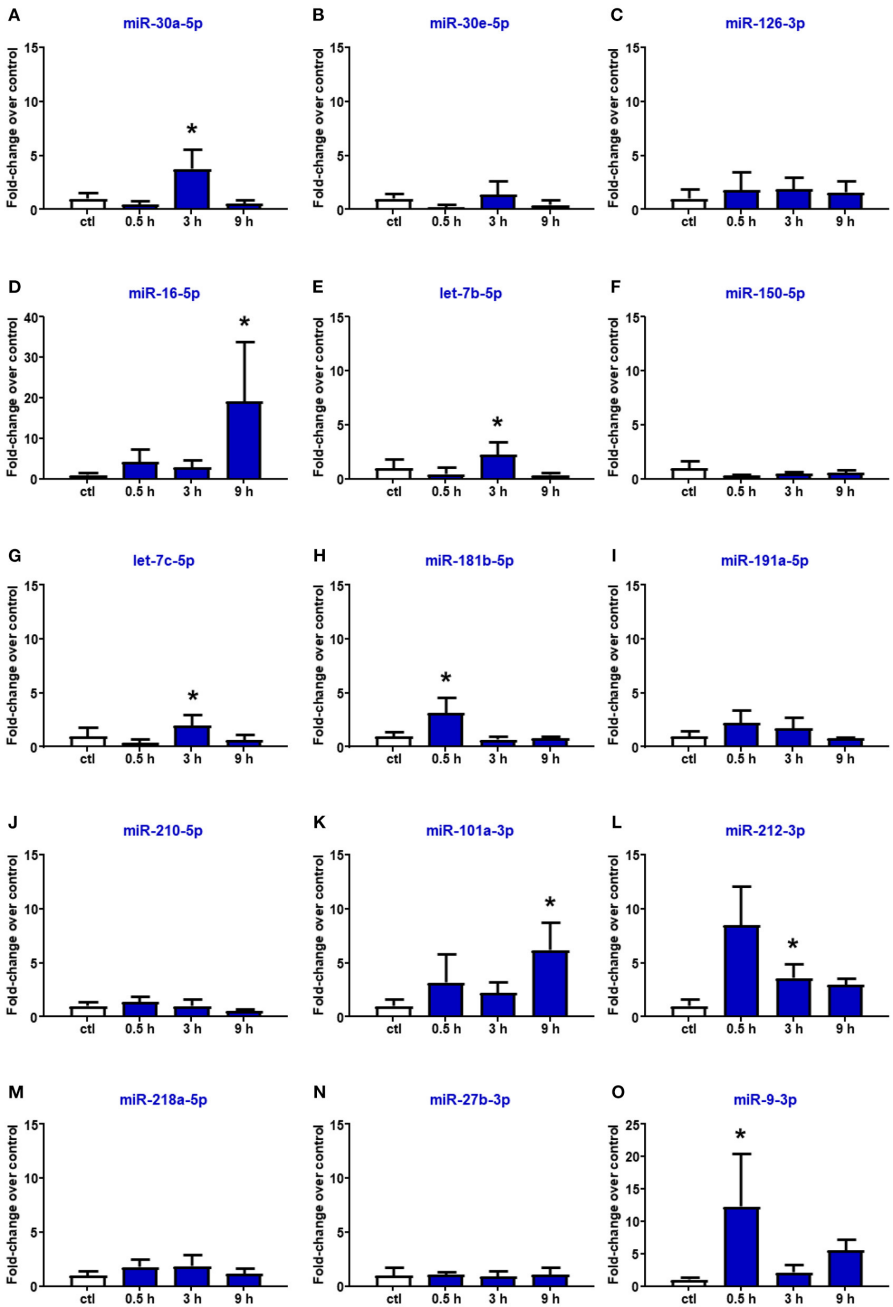
Plasma microRNA expression analysis after transient middle cerebral artery occlusion by real-time PCR. microRNA levels analyzed by real-time PCR in plasma samples withdrawn from the tail vein of rats subjected to tMCAO are expressed as fold change over the control group. **(A–O)** indicate miRNAs evaluated. Each column represents the mean ± SD. The results of the microRNA expression were normalized with respect to U6 snRNA as internal control. *n* = 4 or 5 per group. *p < 0.05 vs. control group.

Reperfusion affected the expression of miR-16-5p, miR-101a-3p, and miR-27b-3p. In particular, in the plasma of rats subjected to tMCAO, the upregulation of miR-16 and miR-101a-3p was delayed compared to the expression levels assessed after pMCAO, showing a statistical increment after 9 h from reperfusion (Figure 4). By contrast, miR-218 and miR-27b resulted to be not modified by tMCAO compared to the control group (Figure 4). Furthermore, miR-30a, let-7b, let-7c, miR-212, and miR-9, whose expression was not modulated by permanent ischemia, were upregulated after reperfusion (Figures 4A,E,G,L,O).

### Information on microRNA–Gene–Disease Ontology Interactions

microRNA–target interaction analysis showed that, among the proteins involved in the control of ionic homeostasis, miR-150-5p activity has been linked, among others, to the control of immune response after stroke. In addition, we identified sodium/calcium exchanger, NCX1, and zinc transporter, ZnT6, and transient receptor potential cation channel, TRPM7, as putative additional targets. All these membrane proteins are implied in cellular ionic homeostasis, and their activity has been strongly linked to stroke pathophysiology (36–38). Similarly, among miR-180 targets linked to stroke pathophysiology, the membrane channel acid sensing ionic channel, ASIC1, and the Na+/H+ exchanger it should be mentioned (Tables 1, 2).

**Table 1:** List of predicted genes as potential targets of circulating microRNAs modulated by ischemic stroke.

	**Sequence**	**Predicted target**
miR-16-5p	5′-UAGCAGCACGUAAAUAUUGGCG-3′	ATP2B2 (ATPase Plasma Membrane Ca^2+^ Transporting 2, PMCA2)
		SLC9A6 (sodium/hydrogen exchanger 6; NHE6)
		SLC9A8 (sodium/hydrogen exchanger 8; NHE8)
		SLC12A2 (sodium-potassium-chloride cotransporter 1, NKCC1)
		SLC24A3 (sodium/potassium/calcium exchanger 3; NCKX3)
		SLC30A8 (zinc transporter, ZnT-8)
		SLC39A9 (zinc transporter, ZIP-9)
		SLC39A10 (zinc transporter, ZIP-10)
miR-101a-3p	5′-UACAGUACUGUGAUAACUGAA-3′	ATP2B2 (ATPase Plasma Membrane Ca^2+^ Transporting 2, PMCA2)
		SLC12A2 (sodium-potassium-chloride cotransporter 1, NKCC1)
		SLC30A6 (zinc transporter, ZnT6)
		SLC30A7 (zinc transporter, ZnT-7)
		SLC39A10 (zinc transporter, ZIP-10)
miR-218a-5p	5′-UUGUGCUUGAUCUAACCAUGU-3′	ASIC1 (acid-sensing ion channel 1, ASIC1)
		SLC12A2 (sodium-potassium-chloride cotransporter 1, NKCC1)
		SLC24A4 (sodium/potassium/calcium exchanger 4; NCKX4)
		SLC39A1 (zinc transporter, ZIP-1)
miR-27b-3p	5′-UUCACAGUGGCUAAGUUCUGC-3′	ASIC1 (acid-sensing ion channel 1, ASIC1)
		ATP2B1 (ATPase Plasma Membrane Ca^2+^ Transporting 1, PMCA1)
		SLC9A4 (sodium/hydrogen exchanger 4; NHE4)
		SLC9A7 (sodium/hydrogen exchanger 7; NHE7)
		SLC24A1 (sodium/potassium/calcium exchanger 1; NCKX1)
		SLC24A4 (sodium/potassium/calcium exchanger 4; NCKX4)
		SLC30A7 (zinc transporter, ZnT-7)
		SLC39A11 (zinc transporter, ZIP-11)
		SLC39A13 (zinc transporter, ZIP-13)

*Only the predicted targets involved in the mechanisms of ionic homeostasis regulation proposed from at least two of the four databases applied are shown*.

**Table 2:** List of predicted genes as potential targets of circulating microRNAs modulated by hemorrhagic stroke.

**microRNA**	**Sequence**	**Predicted target**
miR-150-5p	5′-UCUCCCAACCCUUGUACCAGUG-3′	SLC8A1 (sodium/calcium exchanger 1, NCX1)
		SLC30A5 (zinc transporter, ZnT-5)
		TRPM7 (Transient Receptor Potential Cation Channel M, 7)
let-7b-5p	5′-UGAGGUAGUAGGUUGUGUGGUU-3′	ATP2A2 (ATPase Sarcoplasmic/Endoplasmic Reticulum Ca^2+^ Transporting 2, SERCA2)
		ATP2B4 (ATPase Plasma Membrane Ca2+ Transporting 4, PMCA4)
		SLC8A2 (sodium/calcium exchanger 2, NCX2)
		SLC9A9 (sodium/hydrogen exchanger 9; NHE9)
		SLC30A4 (zinc transporter, ZnT-4)
		TRPM6 (Transient Receptor Potential Cation Channel M, 6)
miR-181b-5p	5′-AACAUUCAUUGCUGUCGGUGGGU-3′	ASIC1 (acid-sensing ion channel 1, ASIC1)
		ATP2A2 (ATPase Sarcoplasmic/Endoplasmic Reticulum Ca^2+^ Transporting 2, SERCA2)
		ATP2B1 (ATPase Plasma Membrane Ca^2+^ Transporting 1, PMCA1)
		ATP2B2 (ATPase Plasma Membrane Ca^2+^ Transporting 2, PMCA2)
		SLC9A6 (sodium/hydrogen exchanger 6; NHE6)
		SLC12A5 (potassium-chloride cotransporter 2, KCC2)
		TRPM3 (Transient Receptor Potential Cation Channel M, 3)
let-7c-5p	5′-UGAGGUAGUAGGUUGUAUGGUU-3′	ATP2A2 (ATPase Sarcoplasmic/Endoplasmic Reticulum Ca^2+^ Transporting 2, SERCA2)
		SLC8A2 (sodium/calcium exchanger 2, NCX2)
		SLC9A9 (sodium/hydrogen exchanger 9; NHE9)
		SLC12A9 (cation-chloride cotransporter 6, CCC6)
		SLC30A4 (zinc transporter, ZnT-4)
		TRPM6 (Transient Receptor Potential Cation Channel M, 6)

*Only the predicted targets involved in the mechanisms of ionic homeostasis regulation proposed from at least two of the four databases applied are shown*.

## Discussion

The present paper, taking advantage of three preclinical animal models of stroke, compared the miRNA blood levels of animals subjected to permanent brain ischemia, transient ischemia, and hemorrhagic stroke at differential time intervals from stroke onset. In particular, starting from a miRNome analysis carried out in the brain tissue of ischemic animals, a list of candidate miRNAs involved in ischemic brain damage was generated. The levels of expression of these miRNAs were then evaluated in the blood of animals subjected to ischemic stroke and compared to those of rats exposed to hemorrhagic stroke.

Our study examined the expression of 548 mirRNAs in the brain tissue of ischemic and sham-operated rats and selected those miRNAs whose expression was increased or reduced after stroke. Therefore, based on the analysis of miRNome in ischemic brain tissue, it has been possible to create a list of miRNAs potentially involved in stroke damage. Since it has been previously demonstrated that the levels of expression of miRNAs in ischemic brain tissue may correlate with those present in the plasma (39), these miRNAs were then evaluated at different time intervals in the blood of rats subjected to ischemic or hemorrhagic stroke. From a first examination that allowed us to narrow our research field on 15 circulating miRNAs examined, four miRNAs—miR-16-5p, miR-101a-3p, miR-218-5p, and miR-27b-3p—were found to be significantly upregulated in the plasma of rats 3 h after pMCAO, whereas four miRNAs—miR-150-5p, let-7b-5p, let 7c-5p, and miR-181b-5p—were selectively upregulated in the plasma of rats subjected to ICH.

These results are of particular relevance since, for the first time, we compared the miRNA levels in the plasma of ischemic animals at different time intervals after the ischemic insult. The choice of the time point examined reflects the time window of intervention in patients affected by stroke. Having a hypothetical biomarker expressed after 6 h from stroke onset is not very helpful in order to properly treat the patient. The time-dependent expression of miRNAs in plasma underlines the importance of a precise timing when miRNAs are considered as prognostic and diagnostic tools. In fact, due to the high stability in biological fluids and the reproducibility of detection, circulating miRNAs have been proposed as new non-invasive biomarkers for the diagnosis of many neurodegenerative disorders, including stroke (40–42).

On the other hand, the importance to have a tool for a rapid diagnosis of stroke represents a medical need since the diagnosis of stroke is remarkably time-consuming, being mainly dependent upon examination by a clinical care provider and by means of various neuroimaging techniques. This way of proceeding leads to fewer possibilities of starting an effective recanalizing therapy in stroke patients within the defined time interval of 4.5 h. Therefore, much effort must be made in order to improve the diagnostic decision-making process. In this direction, the possibility to rely on an easily detectable circulating biomarker able to distinguish between ischemic and hemorrhagic stroke could be of great help for the clinicians. In this context, the role of miRNA is a developing promising field, with growing interest in their potential application as a biomarker for the rapid diagnosis and prognosis of brain ischemia as well as for the development of therapeutic agents (18, 43). Different patient-based studies have already reported some changes in the circulatory expression of miRNA during cerebral ischemia (44).

As anticipated above, miRNAs are present in human plasma or in serum in a remarkably stable form and represent potentially informative biomarkers for a range of diseases. Chen et al. demonstrated that plasma miRNAs are stable and protected against RNases as well as other prohibitive conditions, including boiling, low/high pH, extended storage, and repetitive freezing/thawing cycles (45). The expression levels of miRNAs in blood have been found to be reproducible and indicative of the disease state. Although the mechanism of how miRNAs are released into the circulation is unclear, their presence in the circulation and association with diverse pathophysiological states are now generally accepted. Circulating miRNAs in plasma are altered both qualitatively and quantitatively in a variety of conditions, including different tumor types, cardiovascular diseases, stroke, and neurodegenerative diseases (46).

It is widely believed that miRNAs released from damaged cells or circulating cells lead to increased serum miRNA expression (47). The present study reports unique circulating miRNA expression profiles following cerebral ischemia in adult male rats. In fact, to investigate the time dependency on the expression of stroke-related miRNAs, three time points were evaluated: 0.5, 3, and 9 h. Among the 15 miRNAs considered, miR-101a-3p emerges as the most promising ischemic stroke biomarker for further future evaluation. The levels of expression of miR-101a-3p increase at early time intervals, 0.5 and 3 h, after permanent ischemia induction and at a later time interval, 9 h, after transient ischemia. No changes were detected after hemorrhagic stroke.

As for hemorrhagic stroke, the most promising diagnostic candidate miRNAs are miR-150-5p and miR-181-5p, whose levels of expression in the blood of hemorrhagic rats are selectively upregulated starting from 30 min after hemorrhagic stroke induction.

From *in silico* analysis data and from data that already appeared in the scientific literature, it is possible to link the role of these three miRNAs to stroke pathophysiology.

In fact, miR-101a-3p has one of its major targets in polycomb repressive complex 2, a multi-protein complex including histone methyltransferase enhancer of zeste homolog 2 (48, 49), which mediates gene silencing *via* the tri-methylation of histone 3 at lysine 27 (H3K27me3) (50, 51).

As for miR-150-5p, its activity has been linked, among others, to the control of immune response after stroke. In addition, we identified sodium/calcium exchanger, NCX1, zinc transporter, ZnT6, and transient receptor potential cation channel, TRPM7, as putative additional targets. All these membrane proteins are implied in cellular ionic homeostasis, and their activity has been strongly linked to stroke pathophysiology (36–38). Similarly, among miR-180 targets linked to stroke pathophysiology, the membrane channel acid sensing ionic channel, ASIC1, and the Na^+^/H^+^ exchanger should be mentioned (52, 53).

Collectively, our study identified some peculiar miRNAs expressed in the plasma of hemorrhagic rats and pointed out the importance of a precise time point definition in order to render the use of miRNAs as stroke biomarkers more reliable.

## Limitations

Future studies should include a larger set of animals, eventually of both genders, affected by comorbidities, and at different ages. Furthermore, it is important to underline the need to speed up all those technical procedures capable of detecting miRNAs in the shortest and simplest possible way, i.e., by setting up innovative sensors capable of measuring miRNAs in a few seconds, to avoid running into the same problems currently present with the use of CT and MRI to make a differentiated diagnosis of ischemic and hemorrhagic stroke.

## Data Availability Statement

The datasets presented in this study can be found in online repositories. The names of the repository/repositories and accession number(s) can be found below: GEO Submission (GSE184975) [NCBI tracking system #22375569].

## Ethics Statement

The animal study was reviewed and approved by Ethical Committee of University of Naples Federico II.

## Author Contributions

GP, LA, and PC: conception and design of the study. PC, OC, and MT: acquisition and analysis of data. PC, OC, MT, AV, GP, LA, and MT: drafting a significant portion of the manuscript, tables, and figures. PC and MT evaluated expression of miRNA in blood samples. All authors contributed to the article and approved the submitted version.

## Funding

This work was supported by grants from the Programma Operativo Nazionale PON PERMEDNET (ArSO1_1226) from the Italian Ministry of Research, MIUR, to LA and PON NEON (ARS01_00769) from the Italian Ministry of Research, MIUR, to GP.

## Conflict of Interest

The authors declare that the research was conducted in the absence of any commercial or financial relationships that could be construed as a potential conflict of interest.

## Publisher's Note

All claims expressed in this article are solely those of the authors and do not necessarily represent those of their affiliated organizations, or those of the publisher, the editors and the reviewers. Any product that may be evaluated in this article, or claim that may be made by its manufacturer, is not guaranteed or endorsed by the publisher.

## Abbreviations

rtPA: recombinant tissue plasminogen activator
MCAO: middle cerebral artery occlusion
pMCAO: permanent middle cerebral artery occlusion
tMCAO: transient middle cerebral artery occlusion
ICH: intracerebral collagenase-induced hemorrhage
miRNA: microRNA
DALYs: disability-adjusted life years
CT: computed tomography
MRI: magnetic resonance imaging
HDL: high-density lipoproteins
CBF: cerebral blood flow
PRC2: polycomb repressive complex 2
EZH2: enhancer of zeste homolog 2
H3K27me3: tri-methylation of histone 3 at lysine 27
NCX1: sodium/calcium exchanger 1
ZnT6: zinc transporter 6
TRPM7, transient receptor potential cation channel subfamily M: 7
ASIC1: acid-sensing ionic channel 1.
