# Supplementary material for: Hemorrhagic Stroke Induces a Time-Dependent Upregulation of miR-150-5p and miR-181b-5p in the Bloodstream
Source: Front Neurol. 2021 Oct 27;12:736474. doi: 10.3389/fneur.2021.736474 (PMC8580415; doi:10.3389/fneur.2021.736474)
Supplement: Supplementary file 1 [file Data_Sheet_1.PDF]

	TargetScan	miRDB	miRmap web	PicTar
	ATP2B2 (ATPase Plasma Membrane Ca2+ Transporting 2, PMCA2)	SLC9A6 (sodium/hydrogen exchanger 6; NHE6)	ATP2B2 (ATPase Plasma Membrane Ca2+ Transporting 2, PMCA2)	TRPM2 (Transient Receptor Potential Cation Channel M, 2)
	TRPM3 (Transient Receptor Potential Cation Channel M, 3)	SLC12A2 (sodium-potassium-chloride cotransporter 1, NKCC1)	SLC9A8 (sodium/hydrogen exchanger 8; NHE8)	ATP2B2 (ATPase Plasma Membrane Ca2+ Transporting 2, PMCA2)
	SLC9A6 (sodium/hydrogen exchanger 6; NHE6)	SLC39A10 (zinc transporter, ZIP-10	SLC9A6 (sodium/hydrogen exchanger 6; NHE6)	SLC24A3 (sodium/potassium/calcium exchanger 3; NCKX3)
	SLC9A8 (sodium/hydrogen exchanger 8; NHE8)	SLC39A9 (zinc transporter, ZIP-9)	SLC12A2 (sodium-potassium-chloride cotransporter 1, NKCC1)	SLC12A2 (sodium-potassium-chloride cotransporter 1, NKCC1)
	SLC39A9 (zinc transporter, ZIP-9)	SLC30A8 (zinc transporter, ZNT8)	SLC30A8 (zinc transporter, ZNT8)	SLC9A6 (sodium/hydrogen exchanger 6; NHE6)
	SLC12A2 (sodium-potassium-chloride cotransporter 1, NKCC1)	SLC24A3 (sodium/potassium/calcium exchanger 3; NCKX3)	SLC24A1 (sodium/potassium/calcium exchanger 1; NCKX1)	
	SLC39A10 (zinc transporter, ZIP-10)		SLC12A1 (sodium-potassium-chloride cotransporter 2, NKCC2)	
	SLC24A3 (sodium/potassium/calcium exchanger 3; NCKX3)		SLC39A9 (zinc transporter, ZIP-9)	
			SLC30A7 (zinc transporter, ZNT7)	
			SLC39A10 (zinc transporter, ZIP-10)	
			SLC30A4 (zinc transporter, ZNT4)	
			SLC39A1 (zinc transporter, ZIP-1)	
**miR-101a-3p**	**TargetScan**	**miRDB**	**miRmap web**	**PicTar**
	ASIC1 (acid-sensing ion channel 1, ASIC1)	ATP2B2 (ATPase Plasma Membrane Ca2+ Transporting 2, PMCA2)	ATP2B2 (ATPase Plasma Membrane Ca2+ Transporting 2, PMCA2)	ATP2B2 (ATPase Plasma Membrane Ca2+ Transporting 2, PMCA2)
	ATP2B2 (ATPase Plasma Membrane Ca2+ Transporting 2, PMCA2)	SLC12A2 (sodium-potassium-chloride cotransporter 1, NKCC1)	ATP2B1 (ATPase Plasma Membrane Ca2+ Transporting 1, PMCA1)	SLC12A2 (sodium-potassium-chloride transporter 1, NKCC1)
	SLC12A2 (sodium-potassium-chloride cotransporter 1, NKCC1)	SLC39A10 (zinc transporter, ZIP-10)	SLC12A2 (sodium-potassium-chloride transporter 1, NKCC1)	SLC30A7 (zinc transporter, ZNT7)
	SLC39A10 (zinc transporter, ZIP-10)	SLC30A7 (zinc transporter, ZNT7)	SLC39A10 (zinc transporter, ZIP-10)	
			SLC30A7 (zinc transporter, ZNT7)	
			SLC30A1 (zinc transporter, ZNT1)	
			SLC39A6 (zinc transporter, ZIP-6)	
**miR-218a-5p**	**TargetScan**	**miRDB**	**miRmap web**	**PicTar**
	ASIC1 (acid-sensing ion channel 1, ASIC1)	ASIC1 (acid-sensing ion channel 1, ASIC1)	SLC24A4 (sodium/potassium/calcium exchanger 4; NCKX4)	SLC24A4 (sodium/potassium/calcium exchanger 4; NCKX4)
	SLC24A4 (sodium/potassium/calcium exchanger 4; NCKX4)	SLC39A1 (zinc transporter, ZIP-1)	SLC9A8 (sodium/hydrogen exchanger 8; NHE8)	SLC30A10 (zinc transporter, ZNT10)
	SLC39A1 (zinc transporter, ZIP-1)	SLC24A4 (sodium/potassium/calcium exchanger 4; NCKX4)	SLC39A1 (zinc transporter, ZIP-1)	SLC12A2 (sodium-potassium-chloride cotransporter 1, NKCC1)
	SLC12A2 (sodium-potassium-chloride cotransporter 1, NKCC1)	SLC12A2 (sodium-potassium-chloride cotransporter 1, NKCC1)	SLC12A2 (sodium-potassium-chloride cotransporter 1, NKCC1)	
	SLC39A14 (zinc transporter, ZIP-14)		SLC9A6 (sodium/hydrogen exchanger 6; NHE6)	
			SLC9A2 (sodium/hydrogen exchanger 2; NHE2)	

**miR-27b-3p**	**TargetScan**	**miRDB**	**miRmap web**	**PicTar**
	ATP2B1 (ATPase Plasma Membrane Ca2+ Transporting 1, PMCA1)	ATP2B1 (ATPase Plasma Membrane Ca2+ Transporting 1, PMCA1)	ATP2B1 (ATPase Plasma Membrane Ca2+ Transporting 1, PMCA1)	ATP2B1 (ATPase Plasma Membrane Ca2+ Transporting 1, PMCA1)
	ASIC1 (acid-sensing ion channel 1, ASIC1)	ASIC1 (acid-sensing ion channel 1, ASIC1)	SLC39A11 (zinc transporter, ZIP-11)	SLC39A11 (zinc transporter, ZIP-11)
	SLC39A11 (zinc transporter, ZIP-11)	TRPM3 (Transient Receptor Potential Cation Channel M, 3)	SLC30A1 (zinc transporter, ZNT1)	SLC39A13 (zinc transporter, ZIP-13)
	SLC39A13 (zinc transporter, ZIP-13)	SLC39A11 (zinc transporter, ZIP-11)	SLC9A4 (sodium/hydrogen exchanger 4; NHE4)	
	SLC24A2 (sodium/potassium/calcium exchanger 2; NCKX2)	SLC24A1 (sodium/potassium/calcium exchanger 1; NCKX1)	SLC30A7 (zinc transporter, ZNT7)	
	SLC30A7 (zinc transporter, ZNT7)	SLC30A7 (zinc transporter, ZNT7)	SLC24A1 (sodium/potassium/calcium exchanger 1; NCKX1)	
	SLC9A7 (sodium/hydrogen exchanger 7; NHE7)	SLC24A4 (sodium/potassium/calcium exchanger 4; NCKX4)	SLC39A13 (zinc transporter, ZIP-13)	
	SLC24A4 (sodium/potassium/calcium exchanger 4; NCKX4)	SLC9A4 (sodium/hydrogen exchanger 4; NHE4)		
		SLC9A7 (sodium/hydrogen exchanger 7; NHE7)		
		SLC12A2 (sodium-potassium-chloride cotransporter 1, NKCC1)		
**miR-150-5p**	**TargetScan**	**miRDB**	**miRmap web**	**PicTar**
	SLC30A5 (zinc transporter, ZNT5)	SLC30A5 (zinc transporter, ZNT5)		ATP2B1 (ATPase Plasma Membrane Ca2+ Transporting 1, PMCA1)
		SLC8A1 (sodium/calcium exchanger 1, NCX1)		
		SLC39A7 (zinc transporter, ZIP-7)		
		SLC12A5 (SODIUM-POTASSIUM-CHLORIDE COTRANSPORTER 2, KCC2)		
		SLC39A14 (zinc transporter, ZIP-14)		
		TRPM7 (Transient Receptor Potential Cation Channel M, 7)		
**Let-7b-5p**	**TargetScan**	**miRDB**	**miRmap web**	**PicTar**
	TRPM6 (Transient Receptor Potential Cation Channel M, 6)	SLC30A4 (zinc transporter, ZNT4)	ATP2A2 (ATPase Sarcoplasmic/Endoplasmic Reticulum Ca2+ Transporting 2, SERCA2)	TRPM6 (Transient Receptor Potential Cation Channel M, 6)
	SLC30A6 (zinc transporter, ZNT6)	SLC9A9 (sodium/hydrogen exchanger 9; NHE9)		ATP2A2 (ATPase Sarcoplasmic/Endoplasmic Reticulum Ca2+ Transporting 2, SERCA2)
	SLC9A9 (sodium/hydrogen exchanger 9; NHE9)	SLC8A2 (sodium/calcium exchanger 2, NCX2)		ATP2B1 (ATPase Plasma Membrane Ca2+ Transporting 1, PMCA1)
	SLC30A1 (zinc transporter, ZNT1)	TRPM6 (Transient Receptor Potential Cation Channel M, 6)		SLC30A4 (zinc transporter, ZNT4)
	SLC30A4 (zinc transporter, ZNT4)	ATP2A2 (ATPase Sarcoplasmic/Endoplasmic Reticulum Ca2+ Transporting 2, SERCA2)		SLC9A9 (sodium/hydrogen exchanger 9; NHE9)
	SLC8A2 (sodium/calcium	ATP2B4 (ATPase		

	exchanger 2, NCX2)	Plasma Membrane Ca2+ Transporting 4, PMCA4)		
	SLC30A7 (zinc transporter, ZNT7)			
	ATP2A2 (ATPase Sarcoplasmic/Endoplasmic Reticulum Ca2+ Transporting 2, SERCA2)			
	ATP2B4 (ATPase Plasma Membrane Ca2+ Transporting 4, PMCA4)			
	ATP2B3 (ATPase Plasma Membrane Ca2+ Transporting 3, PMCA3)			
**miR-181b-5p**	**TargetScan**	**miRDB**	**miRmap web**	**PicTar**
	ASIC1 (acid-sensing ion channel 1, ASIC1)	ATP2B1 (ATPase Plasma Membrane Ca2+ Transporting 1, PMCA1)	ATP2B2 (ATPase Plasma Membrane Ca2+ Transporting 2, PMCA2)	ATP2A2 (ATPase Sarcoplasmic/Endoplasmic Reticulum Ca2+ Transporting 2, SERCA2)
	TRPM3 (Transient Receptor Potential Cation Channel M, 3)	ATP2B3 (ATPase Plasma Membrane Ca2+ Transporting 3, PMCA3)		ATP2B2 (ATPase Plasma Membrane Ca2+ Transporting 2, PMCA2)
	TRPM7 (Transient Receptor Potential Cation Channel M, 7)	ATP2B2 (ATPase Plasma Membrane Ca2+ Transporting 2, PMCA2)		SLC9A6 (sodium/hydrogen exchanger 6; NHE6)
	ATP2B1 (ATPase Plasma Membrane Ca2+ Transporting 1, PMCA1)	ATP2A2 (ATPase Sarcoplasmic/Endoplasmic Reticulum Ca2+ Transporting 2, SERCA2)		
	ATP2A2 (ATPase Sarcoplasmic/Endoplasmic Reticulum Ca2+ Transporting 2, SERCA2)	ASIC1 (acid-sensing ion channel 1, ASIC1)		
	ATP2B2 (ATPase Plasma Membrane Ca2+ Transporting 2, PMCA2)	TRPM3 (Transient Receptor Potential Cation Channel M, 3)		
	SLC9A6 (sodium/hydrogen exchanger 6; NHE6)	SLC12A5 (potassium-chloride cotransporter 2, KCC2)		
	SLC8A1 (sodium/calcium exchanger 1, NCX1)	SLC24A3 (sodium/potassium/calcium exchanger 3; NCKX3)		
	SLC30A1 (zinc transporter, ZNT1)			
	SLC9A8 (sodium/hydrogen exchanger 8; NHE8)			
	SLC12A5 (potassium-chloride cotransporter 2, KCC2)			
**Let-7c-5p**	**TargetScan**	**miRDB**	**miRmap web**	**PicTar**
	SLC12A9 (cation-chloride cotransporter 6, CCC6)	TRPM6 (Transient Receptor Potential Cation Channel M, 6)	ATP2A3 (ATPase Sarcoplasmic/Endoplasmic Reticulum Ca2+ Transporting 3, SERCA3)	SLC30A4 (zinc transporter, ZNT4)
	SLC30A6 (zinc transporter, ZNT6)	ATP2A2 (ATPase Sarcoplasmic/Endoplasmic Reticulum Ca2+ Transporting 2, SERCA2)	ATP2A2 (ATPase Sarcoplasmic/Endoplasmic Reticulum Ca2+ Transporting 2, SERCA2)	SLC9A9 (sodium/hydrogen exchanger 9; NHE9)
	SLC9A9 (sodium/hydrogen exchanger 9; NHE9)	ATP2B4 (ATPase Plasma Membrane Ca2+ Transporting 4, PMCA4)		ATP2A2 (ATPase Sarcoplasmic/Endoplasmic Reticulum Ca2+ Transporting 2, SERCA2)
	SLC30A1 (zinc transporter, ZNT1)	SLC30A4 (zinc transporter, ZNT4)		ATP2B1 (ATPase Plasma Membrane Ca2+ Transporting 1, PMCA1)
	SLC30A4 (zinc transporter, ZNT4)	SLC12A9 (cation-chloride cotransporter 6, CCC6)		TRPM6 (Transient Receptor Potential Cation Channel M, 6)
	SLC8A2 (sodium/calcium	SLC9A9		

	exchanger 2, NCX2)	(sodium/hydrogen exchanger 9; NHE9)		
	SLC30A7 (zinc transporter, ZNT7)	SLC8A2 (sodium/calcium exchanger 2, NCX2)		

Supplemental Table1. List of predicted genes as potential targets of analysed circulating microRNAs involved in stroke progression by the regulation of ionic hoemostasis
