# Supplementary figures and images for: Hemorrhagic Stroke Induces a Time-Dependent Upregulation of miR-150-5p and miR-181b-5p in the Bloodstream
Source: Front Neurol. 2021 Oct 27;12:736474. doi: 10.3389/fneur.2021.736474 (PMC8580415; doi:10.3389/fneur.2021.736474)

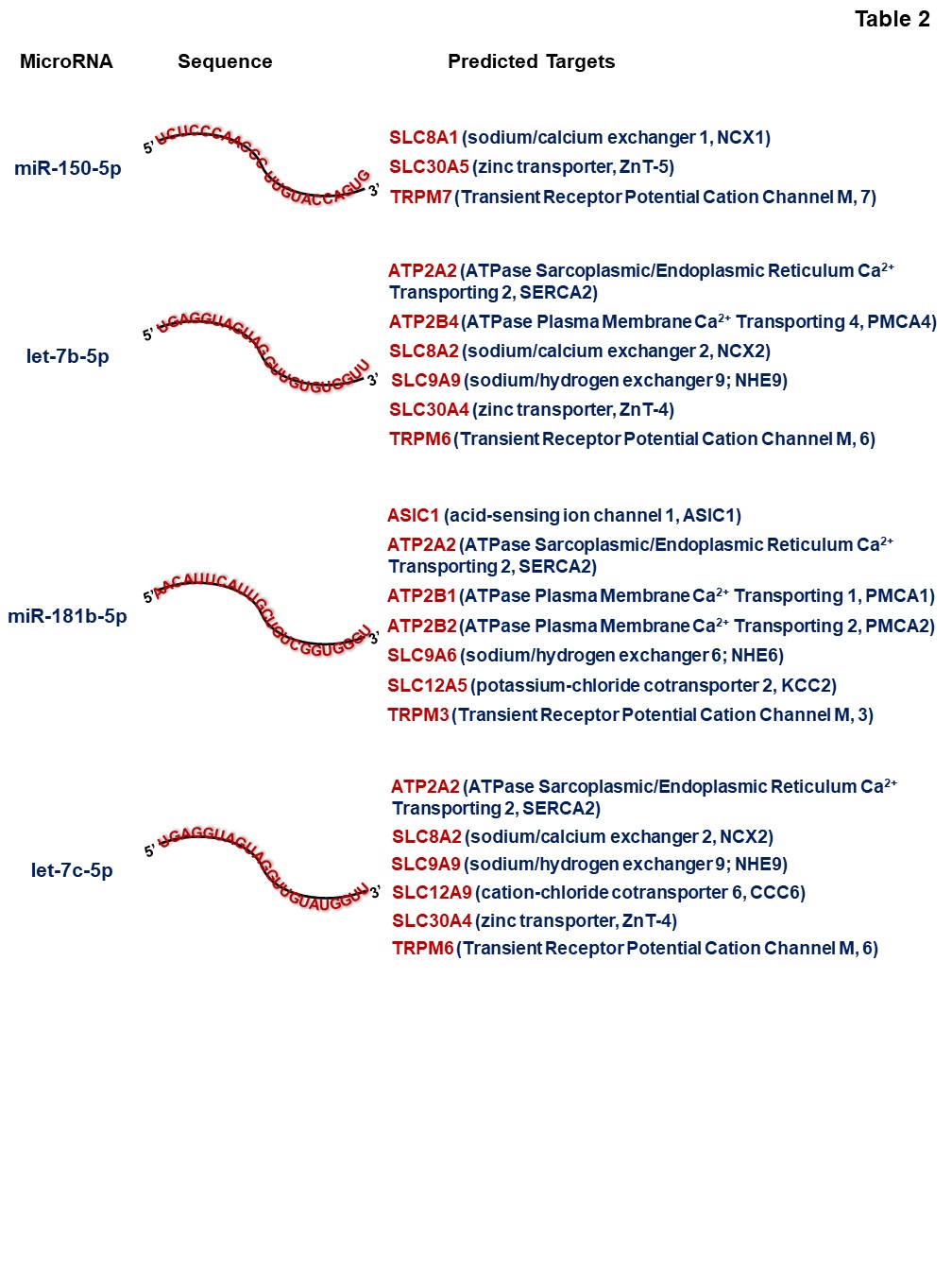

Supplement: Supplementary file 5 [file Image_1.JPEG]

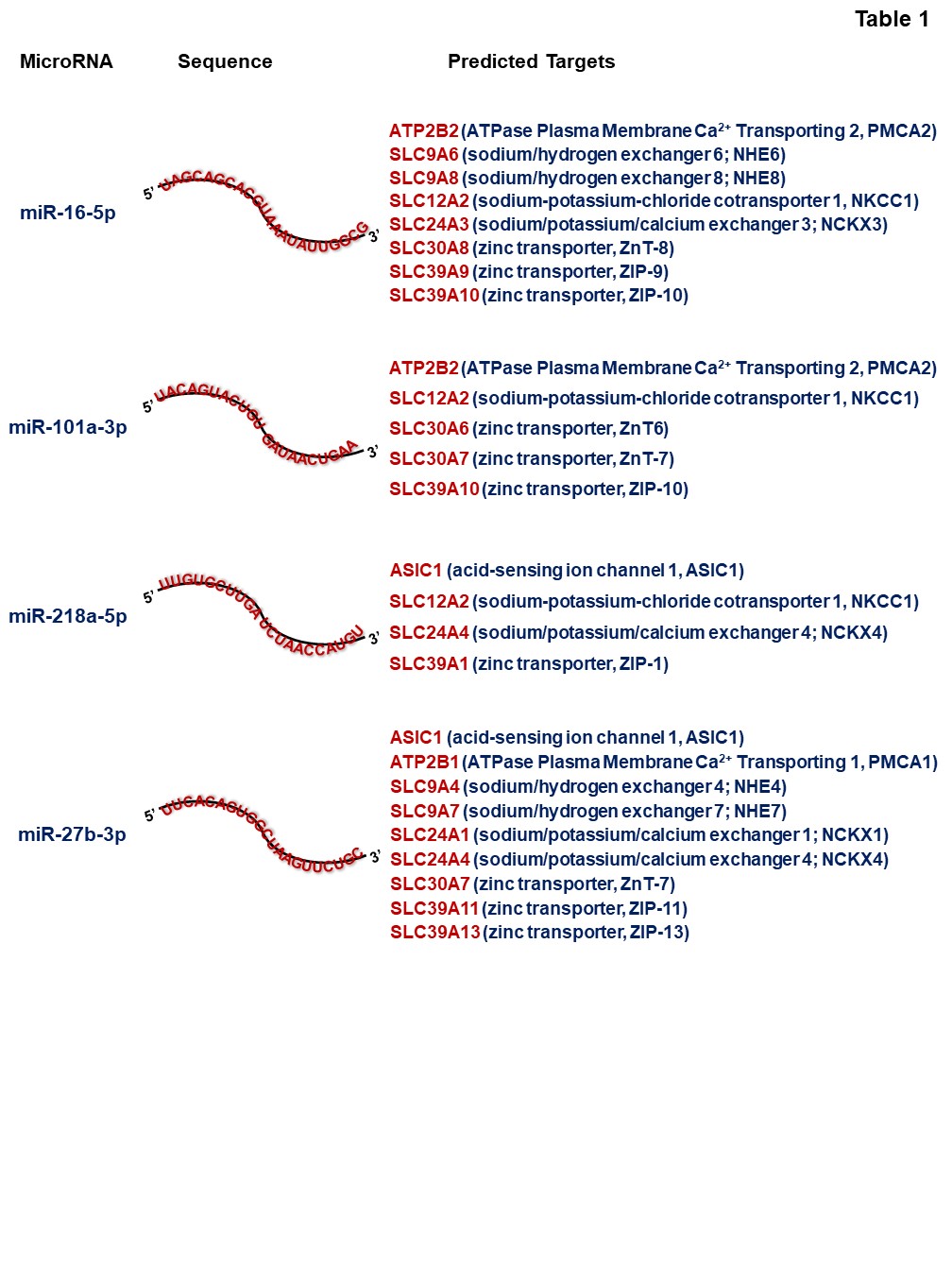

Supplement: Supplementary file 6 [file Image_2.JPEG]
